# More Panel Incidents at Dundee and Wisbech

**Published:** 1914-01-31

**Authors:** 


### More Panel Incidents at Dundee and Wisbech.

Week by week the crop of unfortunate incidents which the panel system appears to foster gets larger and larger. In a sense the Insurance Act is accountable for them, inasmuch as without it the circumstances of panel practice would have been non-existent; but some of them seem to be the kind of results that ensue naturally when men of weak character are afforded the opportunity of receiving more money than they can legitimately earn. This type of abuse is directly encouraged by the panel system, which allows a man to take as many patients as he can get, remunerates him according to their number, and requires no guarantee that his work shall be thoroughly and conscientiously done. In London, as is common knowledge, there are panel doctors with four, five, and even six thousand names on their lists: a scamped service being an inevitable result. In the provinces a larger proportion of the medical practitioners is on the panels, and in most towns and country districts the average number of names on each list is probably smaller than in London. However, there are plenty of cases of panel doctors with over-swollen lists all over the Kingdom, and the problem of reducing them to reasonable dimensions has confronted a good many Insurance Committees.

The muddle is, of course, the fault of the Act and its regulations: and of its author, who was prepared to blink any violation of the spirit of panel service provided he could assert publicly that the panels were filled. At Dundee a good example of the results of this policy has been afforded lately. The Insurance Committee there have had under consideration the limitation of any one panel doctor's list to two thousand insured persons. This rule has been proposed on the express ground that the doctors cannot satisfactorily undertake more than that number. As a matter of fact, probably not one in ten would be justified in taking as many. The medical profession in Dundee have by a large majority approved of the proposed limitation. There are, it appears, only ten practitioners who would be affected by it, and they have so far declined to reduce their panel lists to the required maximum. The mover of a resolution at the meeting of the Committee avowed his desire to persuade these ten to adopt the restriction voluntarily; but the report of the discussion makes it clear that his efforts have not been successful. It is exceedingly doubtful whether an Insurance Committee has any power to enforce its wishes in a matter such as this, supposing the panel doctors in question remain stiff-necked. But the fact that such incidents arise sufficiently proves this weakness of the Act and its resulting consequences of injustice to the insured and demoralisation of the panellers.

### More Troubles at Wisbech.

The small Cambridgeshire town of Wisbech has been the seat of stirring events in connection with National Insurance. It is one of the very few places where the panel was such a hopeless failure that a whole-time medical service was instituted and the panels closed for three years. One of the whole-times who were sent down to inaugurate this service created a sensation eventually by committing suicide after being detected in the sending of scurrilous postcards. Notwithstanding this fact, and the bad record which he had brought with him from London, a "well-arranged Radical riot," as the *Daily Express* calls it, took place after the suicide, directed against the non-panel doctors in the town.

So much for the medical service at Wisbech. Equally distinguished is the record of the local (Isle of Ely) Insurance Committee, an amazing example of which has just come to light. In February of last year an insured Wisbech woman wrote to the Clerk to the Committee to ask for permission to make her own arrangements for medical benefit. She received in return a form of application to be filled in and returned, along with the medical ticket. This was promptly done, and as no more was heard of the matter she concluded that the application had been favourably received. In November this insured person fell ill, was attended by her own private doctor, and sent to the Insurance Committee for a contribution to the bill. She got a letter in reply from the Clerk to the Committee, a solicitor, denying the receipt of her application and refusing in consequence any contribution from Insurance funds.

In reply the insured person wrote that she was able to prove the delivery of the form of application and the medical ticket; but no answer was forthcoming. She then laid the matter before the Insurance Commissioners, who sent the correspondence on to the local Committee. Thus stimulated, the Clerk to the Committee succeeded in finding the missing application, which he had first of all denied all knowledge of. But he proceeded to the astonishing statement that permission to "make own arrangements" for medical benefit could only be granted provided all the three whole-time doctors agreed to it. This statement is, of course, merely an evasion, and an unworthy one at that; and the rest of the dealings of the Committee with the insured person were characterised by an equal want of straightforwardness and fair dealing. It is not very surprising to learn that there is dissatisfaction in the neighbourhood of Ely with the Insurance Act and its local administration.

### A Nice Point.

A curious point has arisen in connection with the conditions attached to the staff vacancies at King Edward VII.'s Hospital, Cardiff. The advertisement said that "no member of the staff should hold any . . . panel appointment." Among the candidates were two who did hold such appointments, but were apparently willing to resign them if elected. Others similarly situated did not apply because they thought themselves ineligible; and the question has been taken up whether or not these particular entries do or do not comply with the rules.

